# 2-(3-Morpholino­prop­yl)-2,3-dihydro-1*H*-pyrrolo­[3,4-*b*]quinolin-1-one monohydrate

**DOI:** 10.1107/S1600536810046349

**Published:** 2010-11-13

**Authors:** Yu-Hua Long, Ting Zhou, Ding-Qiao Yang, Wen-Ling Wang, Han-Mei Zhang

**Affiliations:** a School of Chemistry and Environment, South China Normal University, Guangzhou 510006, People’s Republic of China

## Abstract

In the title compound, C_18_H_21_N_3_O_2_·H_2_O, the fused-ring system is approximately planar [maximum atomic deviation = 0.028 (3) Å]; the morpholine ring displays a chair conformation. The crystal packing is stabilized by classical inter­molecular O—H⋯O and O—H⋯N hydrogen bonds and weak C—H⋯O hydrogen bonds between the organic mol­ecules and the water mol­ecules.

## Related literature

For the properties and biological activity of quinoline deriv­atives, see: Vaitilingam *et al.* (2004 ▶); Lee *et al.* (2004 ▶); Zwaagstra *et al.* (1998 ▶); Roma *et al.* (2000 ▶); Ferrarini *et al.* (2000 ▶). For the preparation of quinoline derivatives, see: Zhou *et al.* (2010 ▶); Yang *et al.* (2008 ▶).
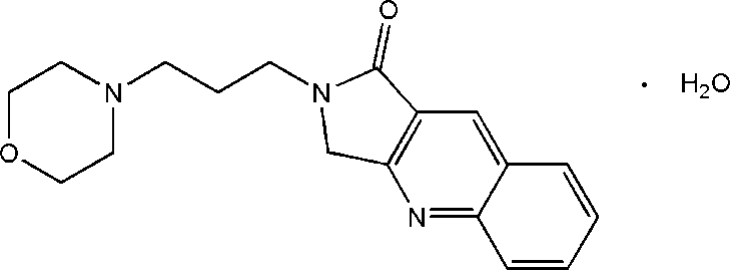


## Experimental

### 

#### Crystal data

C_18_H_21_N_3_O_2_·H_2_O
*M* *_r_* = 329.39
Orthorhombic, 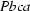

*a* = 7.0107 (16) Å
*b* = 12.655 (3) Å
*c* = 37.609 (9) Å
*V* = 3336.7 (13) Å^3^
*Z* = 8
Mo *K*α radiation
μ = 0.09 mm^−1^
*T* = 296 K
0.30 × 0.28 × 0.27 mm

#### Data collection

Bruker SMART 1000 CCD area-detector diffractometer
15458 measured reflections
2943 independent reflections
1864 reflections with *I* > 2σ(*I*)
*R*_int_ = 0.067

#### Refinement

*R*[*F* ^2^ > 2σ(*F* ^2^)] = 0.057
*wR*(*F* ^2^) = 0.144
*S* = 1.04
2943 reflections
225 parameters
3 restraints
H atoms treated by a mixture of independent and constrained refinement
Δρ_max_ = 0.28 e Å^−3^
Δρ_min_ = −0.41 e Å^−3^

### 

Data collection: *SMART* (Siemens, 1996 ▶); cell refinement: *SAINT* (Siemens, 1996 ▶); data reduction: *SAINT*; program(s) used to solve structure: *SHELXTL* (Sheldrick, 2008 ▶); program(s) used to refine structure: *SHELXTL*; molecular graphics: *SHELXTL*; software used to prepare material for publication: *SHELXTL*.

## Supplementary Material

Crystal structure: contains datablocks global, I. DOI: 10.1107/S1600536810046349/xu5085sup1.cif

Structure factors: contains datablocks I. DOI: 10.1107/S1600536810046349/xu5085Isup2.hkl

Additional supplementary materials:  crystallographic information; 3D view; checkCIF report

## Figures and Tables

**Table 1:** Hydrogen-bond geometry (Å, °)

*D*—H⋯*A*	*D*—H	H⋯*A*	*D*⋯*A*	*D*—H⋯*A*
O1*W*—H1*W*⋯N3^i^	0.86 (4)	2.15 (4)	2.961 (4)	155 (4)
O1*W*—H2*W*⋯O1^ii^	0.87 (4)	1.98 (4)	2.843 (3)	174 (4)
C11—H11*B*⋯O1*W*	0.97	2.47	3.326 (4)	147

Symmetry codes: (i) 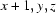; (ii) 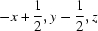.

**Fig. 1.**
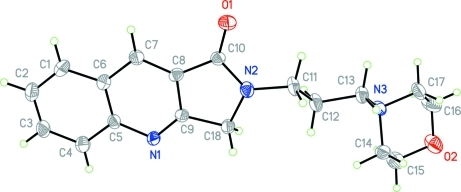
Molecular structure of the title compound showing atom-labelling scheme. Displacement ellipsoids are drawn at the 50% probability level.

**Fig. 2.**
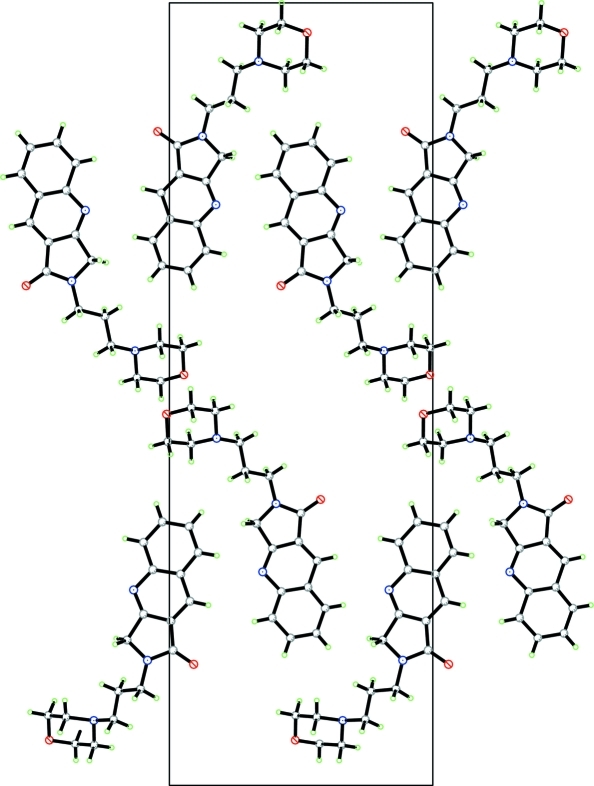
Packing diagram of the title compound.

**Fig. 3.**
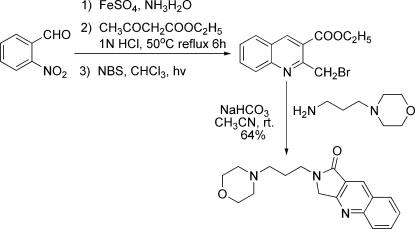
Reaction scheme for the title compound.

## supplementary crystallographic information

### Comment

Quinoline analogues have been reported to display promising antibacterial (Vaitilingam *et al.*, 2004), an-ticancer and antiplatelet (Lee *et al.*, 2004), antiasthmatic (Zwaagstra *et al.*, 1998), antiinflammatory  (Roma *et al.*, 2000), and antihypertensive activities (Ferrarini *et al.*, 2000). We have synthesized some new quinoline derivatives (Yang *et al.*, 2008). In continuation of our efforts to develop  quinoline derivatives with a new structure-activity relationship, herein, we report the synthesis and structure determination the title compound.

The molecular geometry and the atom-labeling scheme of the title compound is illustrated in Fig. 1. The molecule contains three approximately coplanar rings and the dihedral angle between the three rings 1.60 (2)° and 1.20 (5)°, respectively; the C—N2—C—C torsion angles are 43.59° and -137.51°; the morpholine ring shows a stable chair conformation. The crystal structure can be depicted as layers along a-axis which ring systems are parallel to one another. The crystal packing is stabilized by intermolecular interactions between O and H atoms [C—H···O = 2.638Å].

### Experimental

The precursor, ethyl 2-(bromomethyl)quinoline-3-carboxylate, was prepared according to the literature procedure (Yang *et al.*, 2008; Zhou *et al.*, 2010). The title compoud was synthesized by treating 1 mmol of ethyl 2-(bromomethyl)quinoline-3-carboxylate with 1.2 mmol of 3-morpholinopropan-1-amine in the presence of NaHCO_3_ in acetonitrile. The reaction was carried out under the stirring at room temperature for 10 h. Once the reaction was complete, the solid salt was filtered off and the filtrate was then concentrated under reduced pressure. The crude product was purified by silica gel column chromatography with the mixture of methanol and ethyl acetate (v /v = 1/20) to afford the white product. Crystals suitable for X-ray analysis were obtained by slow evaporation of the solution of petroleum ether and dichloromethane, in which the small amount of water was not removed.

### Refinement

Water H atoms were located in a difference Fourier map and refined isotropically. Other H atoms were positioned geometrically and allowed to ride on their parent atoms, with C—H = 0.93–0.97 Å and U_iso_(H) = 1.2U_eq_(C).

### Crystal data

**Table:**

C_18_H_21_N_3_O_2_·H_2_O	*F*(000) = 1408.0
*M**_r_* = 329.39	*D*_x_ = 1.311 Mg m^−^^3^
Orthorhombic, *P**b**c**a*	Mo *K*α radiation, λ = 0.71073 Å
Hall symbol: -P 2ac 2ab	Cell parameters from 1868 reflections
*a* = 7.0107 (16) Å	θ = 3.1–20.4°
*b* = 12.655 (3) Å	µ = 0.09 mm^−^^1^
*c* = 37.609 (9) Å	*T* = 296 K
*V* = 3336.7 (13) Å^3^	Block, colorless
*Z* = 8	0.30 × 0.28 × 0.27 mm

### Data collection

**Table:**

Bruker SMART 1000 CCD area-detector diffractometer	1864 reflections with *I* > 2σ(*I*)
Radiation source: fine-focus sealed tube	*R*_int_ = 0.067
graphite	θ_max_ = 25.0°, θ_min_ = 2.2°
φ and ω scans	*h* = −8→7
15458 measured reflections	*k* = −15→14
2943 independent reflections	*l* = −44→41

### Refinement

**Table:**

Refinement on *F*^2^	Primary atom site location: structure-invariant direct methods
Least-squares matrix: full	Secondary atom site location: difference Fourier map
*R*[*F*^2^ > 2σ(*F*^2^)] = 0.057	Hydrogen site location: inferred from neighbouring sites
*wR*(*F*^2^) = 0.144	H atoms treated by a mixture of independent and constrained refinement
*S* = 1.04	*w* = 1/[σ^2^(*F*_o_^2^) + (0.0611*P*)^2^ + 1.0475*P*] where *P* = (*F*_o_^2^ + 2*F*_c_^2^)/3
2943 reflections	(Δ/σ)_max_ < 0.001
225 parameters	Δρ_max_ = 0.28 e Å^−^^3^
3 restraints	Δρ_min_ = −0.41 e Å^−^^3^

### Special details

**Table:**

Geometry. All esds (except the esd in the dihedral angle between two l.s. planes) are estimated using the full covariance matrix. The cell esds are taken into account individually in the estimation of esds in distances, angles and torsion angles; correlations between esds in cell parameters are only used when they are defined by crystal symmetry. An approximate (isotropic) treatment of cell esds is used for estimating esds involving l.s. planes.
Refinement. Refinement of F^2^ against ALL reflections. The weighted R-factor wR and goodness of fit S are based on F^2^, conventional R-factors R are based on F, with F set to zero for negative F^2^. The threshold expression of F^2^ > 2sigma(F^2^) is used only for calculating R-factors(gt) etc. and is not relevant to the choice of reflections for refinement. R-factors based on F^2^ are statistically about twice as large as those based on F, and R- factors based on ALL data will be even larger.

### Fractional atomic coordinates and isotropic or equivalent isotropic displacement parameters (Å^2^)

**Table:**

	*x*	*y*	*z*	*U*_iso_*/*U*_eq_	
N1	0.1806 (3)	0.84470 (14)	0.25789 (5)	0.0447 (5)	
O1	0.1534 (3)	1.07919 (12)	0.35666 (4)	0.0547 (5)	
N2	0.1728 (3)	0.89892 (13)	0.35114 (5)	0.0406 (5)	
N3	−0.1048 (3)	0.66704 (14)	0.44146 (5)	0.0431 (5)	
O2	−0.2365 (3)	0.47670 (15)	0.47362 (5)	0.0807 (7)	
C6	0.1586 (3)	1.02874 (17)	0.23807 (6)	0.0401 (6)	
C5	0.1701 (3)	0.91850 (17)	0.23121 (6)	0.0402 (6)	
C4	0.1701 (4)	0.8838 (2)	0.19565 (6)	0.0509 (7)	
H4	0.1744	0.8119	0.1907	0.061*	
C1	0.1522 (4)	1.09912 (19)	0.20898 (7)	0.0519 (7)	
H1	0.1446	1.1714	0.2132	0.062*	
C3	0.1638 (4)	0.9544 (2)	0.16832 (7)	0.0561 (7)	
H3	0.1642	0.9301	0.1450	0.067*	
C2	0.1569 (4)	1.0629 (2)	0.17496 (7)	0.0582 (7)	
H2	0.1554	1.1104	0.1561	0.070*	
C9	0.1791 (3)	0.88333 (16)	0.29006 (6)	0.0386 (6)	
C8	0.1644 (3)	0.99006 (16)	0.29942 (5)	0.0371 (6)	
C10	0.1624 (3)	0.99828 (17)	0.33838 (6)	0.0399 (6)	
C7	0.1545 (3)	1.06426 (17)	0.27334 (6)	0.0416 (6)	
H7	0.1454	1.1358	0.2787	0.050*	
C11	0.1741 (4)	0.87224 (18)	0.38875 (6)	0.0445 (6)	
H11A	0.1305	0.9325	0.4025	0.053*	
H11B	0.3034	0.8560	0.3961	0.053*	
C13	0.0266 (4)	0.75468 (17)	0.43519 (6)	0.0443 (6)	
H13A	0.1507	0.7370	0.4450	0.053*	
H13B	−0.0197	0.8171	0.4474	0.053*	
C12	0.0472 (4)	0.77880 (18)	0.39619 (6)	0.0454 (6)	
H12A	0.0990	0.7172	0.3843	0.054*	
H12B	−0.0781	0.7924	0.3863	0.054*	
C15	−0.1663 (5)	0.4788 (2)	0.43826 (7)	0.0720 (9)	
H15A	−0.2717	0.4900	0.4220	0.086*	
H15B	−0.1090	0.4111	0.4327	0.086*	
C17	−0.1709 (5)	0.6639 (2)	0.47821 (7)	0.0656 (9)	
H17A	−0.2290	0.7310	0.4844	0.079*	
H17B	−0.0636	0.6521	0.4940	0.079*	
C14	−0.0214 (4)	0.56444 (18)	0.43321 (7)	0.0547 (7)	
H14A	0.0871	0.5517	0.4486	0.066*	
H14B	0.0234	0.5641	0.4088	0.066*	
C16	−0.3143 (5)	0.5764 (2)	0.48256 (9)	0.0875 (12)	
H16A	−0.3581	0.5748	0.5070	0.105*	
H16B	−0.4237	0.5905	0.4675	0.105*	
C18	0.1877 (4)	0.81867 (18)	0.32341 (6)	0.0467 (6)	
H18A	0.0826	0.7690	0.3246	0.056*	
H18B	0.3072	0.7803	0.3251	0.056*	
O1W	0.5672 (4)	0.72672 (19)	0.39612 (10)	0.1179 (11)	
H1W	0.638 (6)	0.695 (3)	0.4116 (10)	0.17 (2)*	
H2W	0.506 (6)	0.678 (3)	0.3845 (11)	0.20 (2)*	

### Atomic displacement parameters (Å^2^)

**Table:**

	*U*^11^	*U*^22^	*U*^33^	*U*^12^	*U*^13^	*U*^23^
N1	0.0601 (15)	0.0386 (10)	0.0354 (11)	0.0044 (10)	−0.0005 (9)	0.0000 (8)
O1	0.0741 (14)	0.0385 (9)	0.0515 (10)	−0.0030 (8)	0.0045 (9)	−0.0100 (8)
N2	0.0512 (13)	0.0360 (10)	0.0346 (10)	−0.0028 (9)	0.0013 (9)	−0.0007 (8)
N3	0.0506 (13)	0.0402 (11)	0.0384 (11)	−0.0036 (9)	0.0092 (9)	−0.0022 (8)
O2	0.120 (2)	0.0528 (12)	0.0695 (13)	−0.0217 (12)	0.0372 (13)	−0.0008 (9)
C6	0.0372 (15)	0.0409 (13)	0.0424 (13)	−0.0019 (11)	−0.0023 (11)	0.0071 (10)
C5	0.0361 (14)	0.0451 (14)	0.0393 (13)	0.0026 (11)	−0.0004 (11)	0.0037 (10)
C4	0.0583 (18)	0.0533 (15)	0.0410 (14)	0.0107 (13)	−0.0017 (12)	0.0002 (12)
C1	0.0547 (18)	0.0467 (14)	0.0543 (16)	−0.0010 (12)	−0.0034 (13)	0.0114 (12)
C3	0.0527 (18)	0.0750 (19)	0.0406 (14)	0.0123 (14)	−0.0010 (12)	0.0058 (13)
C2	0.0565 (19)	0.0665 (18)	0.0517 (17)	0.0026 (14)	−0.0005 (14)	0.0192 (13)
C9	0.0429 (15)	0.0352 (12)	0.0378 (13)	0.0001 (10)	0.0004 (11)	0.0005 (10)
C8	0.0382 (14)	0.0332 (12)	0.0400 (13)	−0.0036 (10)	0.0025 (11)	−0.0001 (9)
C10	0.0389 (14)	0.0363 (13)	0.0444 (14)	−0.0037 (10)	0.0019 (11)	−0.0029 (10)
C7	0.0445 (16)	0.0338 (12)	0.0465 (14)	−0.0035 (11)	−0.0017 (12)	−0.0003 (10)
C11	0.0495 (16)	0.0495 (14)	0.0346 (13)	−0.0068 (12)	−0.0018 (11)	0.0005 (10)
C13	0.0513 (16)	0.0429 (13)	0.0387 (13)	−0.0055 (12)	−0.0027 (11)	0.0000 (10)
C12	0.0519 (17)	0.0450 (14)	0.0393 (13)	−0.0069 (12)	−0.0013 (11)	0.0015 (10)
C15	0.096 (3)	0.0501 (16)	0.070 (2)	−0.0123 (16)	0.0266 (18)	−0.0086 (14)
C17	0.091 (2)	0.0552 (16)	0.0503 (16)	−0.0108 (16)	0.0242 (15)	−0.0076 (12)
C14	0.068 (2)	0.0441 (14)	0.0522 (15)	0.0011 (13)	0.0158 (14)	0.0000 (11)
C16	0.114 (3)	0.062 (2)	0.086 (2)	−0.0226 (19)	0.054 (2)	−0.0117 (16)
C18	0.0626 (18)	0.0372 (13)	0.0402 (13)	−0.0027 (12)	0.0009 (12)	−0.0006 (10)
O1W	0.091 (2)	0.0689 (15)	0.194 (3)	0.0150 (14)	−0.066 (2)	−0.0318 (18)

### Geometric parameters (Å, °)

**Table:**

N1—C9	1.305 (3)	C8—C10	1.469 (3)
N1—C5	1.373 (3)	C7—H7	0.9300
O1—C10	1.235 (3)	C11—C12	1.506 (3)
N2—C10	1.348 (3)	C11—H11A	0.9700
N2—C11	1.454 (3)	C11—H11B	0.9700
N2—C18	1.460 (3)	C13—C12	1.505 (3)
N3—C14	1.458 (3)	C13—H13A	0.9700
N3—C17	1.458 (3)	C13—H13B	0.9700
N3—C13	1.461 (3)	C12—H12A	0.9700
O2—C15	1.418 (3)	C12—H12B	0.9700
O2—C16	1.415 (4)	C15—C14	1.498 (4)
C6—C7	1.401 (3)	C15—H15A	0.9700
C6—C1	1.411 (3)	C15—H15B	0.9700
C6—C5	1.421 (3)	C17—C16	1.505 (4)
C5—C4	1.408 (3)	C17—H17A	0.9700
C4—C3	1.362 (3)	C17—H17B	0.9700
C4—H4	0.9300	C14—H14A	0.9700
C1—C2	1.360 (3)	C14—H14B	0.9700
C1—H1	0.9300	C16—H16A	0.9700
C3—C2	1.397 (4)	C16—H16B	0.9700
C3—H3	0.9300	C18—H18A	0.9700
C2—H2	0.9300	C18—H18B	0.9700
C9—C8	1.400 (3)	O1W—H1W	0.86 (4)
C9—C18	1.499 (3)	O1W—H2W	0.87 (4)
C8—C7	1.360 (3)		
			
C9—N1—C5	114.99 (18)	N3—C13—C12	111.82 (18)
C10—N2—C11	124.29 (19)	N3—C13—H13A	109.3
C10—N2—C18	113.48 (18)	C12—C13—H13A	109.3
C11—N2—C18	122.21 (17)	N3—C13—H13B	109.3
C14—N3—C17	107.80 (19)	C12—C13—H13B	109.3
C14—N3—C13	112.86 (19)	H13A—C13—H13B	107.9
C17—N3—C13	111.95 (18)	C11—C12—C13	113.39 (19)
C15—O2—C16	109.9 (2)	C11—C12—H12A	108.9
C7—C6—C1	122.1 (2)	C13—C12—H12A	108.9
C7—C6—C5	119.21 (19)	C11—C12—H12B	108.9
C1—C6—C5	118.7 (2)	C13—C12—H12B	108.9
N1—C5—C4	118.8 (2)	H12A—C12—H12B	107.7
N1—C5—C6	122.6 (2)	O2—C15—C14	111.6 (2)
C4—C5—C6	118.6 (2)	O2—C15—H15A	109.3
C3—C4—C5	120.8 (2)	C14—C15—H15A	109.3
C3—C4—H4	119.6	O2—C15—H15B	109.3
C5—C4—H4	119.6	C14—C15—H15B	109.3
C2—C1—C6	121.1 (2)	H15A—C15—H15B	108.0
C2—C1—H1	119.5	N3—C17—C16	109.6 (2)
C6—C1—H1	119.5	N3—C17—H17A	109.8
C4—C3—C2	120.7 (2)	C16—C17—H17A	109.8
C4—C3—H3	119.6	N3—C17—H17B	109.8
C2—C3—H3	119.6	C16—C17—H17B	109.8
C1—C2—C3	120.0 (2)	H17A—C17—H17B	108.2
C1—C2—H2	120.0	N3—C14—C15	110.2 (2)
C3—C2—H2	120.0	N3—C14—H14A	109.6
N1—C9—C8	126.5 (2)	C15—C14—H14A	109.6
N1—C9—C18	124.83 (19)	N3—C14—H14B	109.6
C8—C9—C18	108.63 (18)	C15—C14—H14B	109.6
C7—C8—C9	119.3 (2)	H14A—C14—H14B	108.1
C7—C8—C10	132.08 (19)	O2—C16—C17	111.9 (3)
C9—C8—C10	108.65 (18)	O2—C16—H16A	109.2
O1—C10—N2	125.3 (2)	C17—C16—H16A	109.2
O1—C10—C8	127.9 (2)	O2—C16—H16B	109.2
N2—C10—C8	106.78 (18)	C17—C16—H16B	109.2
C8—C7—C6	117.4 (2)	H16A—C16—H16B	107.9
C8—C7—H7	121.3	N2—C18—C9	102.43 (17)
C6—C7—H7	121.3	N2—C18—H18A	111.3
N2—C11—C12	111.07 (18)	C9—C18—H18A	111.3
N2—C11—H11A	109.4	N2—C18—H18B	111.3
C12—C11—H11A	109.4	C9—C18—H18B	111.3
N2—C11—H11B	109.4	H18A—C18—H18B	109.2
C12—C11—H11B	109.4	H1W—O1W—H2W	107 (4)
H11A—C11—H11B	108.0		
			
C9—N1—C5—C4	−179.8 (2)	C7—C8—C10—N2	179.0 (3)
C9—N1—C5—C6	0.1 (3)	C9—C8—C10—N2	−0.9 (3)
C7—C6—C5—N1	−1.1 (4)	C9—C8—C7—C6	0.4 (3)
C1—C6—C5—N1	178.7 (2)	C10—C8—C7—C6	−179.5 (2)
C7—C6—C5—C4	178.7 (2)	C1—C6—C7—C8	−179.0 (2)
C1—C6—C5—C4	−1.4 (3)	C5—C6—C7—C8	0.8 (3)
N1—C5—C4—C3	−178.7 (2)	C10—N2—C11—C12	137.0 (2)
C6—C5—C4—C3	1.5 (4)	C18—N2—C11—C12	−44.5 (3)
C7—C6—C1—C2	179.8 (2)	C14—N3—C13—C12	75.8 (3)
C5—C6—C1—C2	0.0 (4)	C17—N3—C13—C12	−162.4 (2)
C5—C4—C3—C2	−0.1 (4)	N2—C11—C12—C13	−173.9 (2)
C6—C1—C2—C3	1.5 (4)	N3—C13—C12—C11	177.1 (2)
C4—C3—C2—C1	−1.4 (4)	C16—O2—C15—C14	56.8 (4)
C5—N1—C9—C8	1.3 (4)	C14—N3—C17—C16	−58.6 (3)
C5—N1—C9—C18	179.5 (2)	C13—N3—C17—C16	176.7 (2)
N1—C9—C8—C7	−1.6 (4)	C17—N3—C14—C15	59.0 (3)
C18—C9—C8—C7	180.0 (2)	C13—N3—C14—C15	−176.9 (2)
N1—C9—C8—C10	178.3 (2)	O2—C15—C14—N3	−59.0 (3)
C18—C9—C8—C10	−0.2 (3)	C15—O2—C16—C17	−57.1 (4)
C11—N2—C10—O1	0.3 (4)	N3—C17—C16—O2	59.1 (4)
C18—N2—C10—O1	−178.3 (2)	C10—N2—C18—C9	−1.7 (3)
C11—N2—C10—C8	−179.8 (2)	C11—N2—C18—C9	179.7 (2)
C18—N2—C10—C8	1.6 (3)	N1—C9—C18—N2	−177.5 (2)
C7—C8—C10—O1	−1.1 (5)	C8—C9—C18—N2	1.0 (3)
C9—C8—C10—O1	179.0 (2)		

### Hydrogen-bond geometry (Å, °)

**Table:**

*D*—H···*A*	*D*—H	H···*A*	*D*···*A*	*D*—H···*A*
O1W—H1W···N3^i^	0.86 (4)	2.15 (4)	2.961 (4)	155 (4)
O1W—H2W···O1^ii^	0.87 (4)	1.98 (4)	2.843 (3)	174 (4)
C11—H11B···O1W	0.97	2.47	3.326 (4)	147

Symmetry codes: (i) *x*+1, *y*, *z*; (ii) −*x*+1/2, *y*−1/2, *z*.
